# Supplementary material for: Modeling glioblastoma heterogeneity as a dynamic network of cell states
Source: Mol Syst Biol. 2021 Sep 16;17(9):e10105. doi: 10.15252/msb.202010105 (PMC8444284; doi:10.15252/msb.202010105)
Supplement: Supplementary file 6 — Source Data for Figure 5 [file MSB-17-e10105-s004.zip › Figure5A_sourcedata/GSEA_3017/hallmarks_stateF.GseaPreranked.1621934628066/HALLMARK_APOPTOSIS.html]

Details for gene set HALLMARK\_APOPTOSIS[GSEA]

|  || Dataset | state33017 |
| Phenotype | NoPhenotypeAvailable |
| Upregulated in class | na\_pos |
| GeneSet | HALLMARK\_APOPTOSIS |
| Enrichment Score (ES) | 0.43169075 |
| Normalized Enrichment Score (NES) | 2.1352909 |
| Nominal p-value | 0.0 |
| FDR q-value | 0.0021364167 |
| FWER p-Value | 0.015 |
Table: GSEA Results Summary

  

Fig 1: Enrichment plot: HALLMARK\_APOPTOSIS      
 Profile of the Running ES Score & Positions of GeneSet Members on the Rank Ordered List

  

| PROBE | GENE SYMBOL | GENE\_TITLE | RANK IN GENE LIST | RANK METRIC SCORE | RUNNING ES | CORE ENRICHMENT || 1 | CDKN1A |  |  | 4 | 2.089 | 0.0760 | Yes |
| 2 | CLU |  |  | 5 | 1.852 | 0.1449 | Yes |
| 3 | LGALS3 |  |  | 16 | 1.261 | 0.1873 | Yes |
| 4 | IFITM3 |  |  | 38 | 0.987 | 0.2146 | Yes |
| 5 | SAT1 |  |  | 45 | 0.957 | 0.2475 | Yes |
| 6 | BAX |  |  | 50 | 0.933 | 0.2804 | Yes |
| 7 | SQSTM1 |  |  | 54 | 0.914 | 0.3131 | Yes |
| 8 | APP |  |  | 95 | 0.759 | 0.3232 | Yes |
| 9 | FDXR |  |  | 120 | 0.716 | 0.3390 | Yes |
| 10 | BTG2 |  |  | 124 | 0.708 | 0.3640 | Yes |
| 11 | FEZ1 |  |  | 133 | 0.691 | 0.3861 | Yes |
| 12 | HMOX1 |  |  | 142 | 0.684 | 0.4079 | Yes |
| 13 | CD44 |  |  | 191 | 0.600 | 0.4086 | Yes |
| 14 | TIMP2 |  |  | 196 | 0.590 | 0.4287 | Yes |
| 15 | MMP2 |  |  | 357 | 0.485 | 0.3745 | Yes |
| 16 | PDCD4 |  |  | 360 | 0.484 | 0.3916 | Yes |
| 17 | DPYD |  |  | 383 | 0.477 | 0.3994 | Yes |
| 18 | TGFB2 |  |  | 390 | 0.474 | 0.4143 | Yes |
| 19 | DDIT3 |  |  | 447 | 0.451 | 0.4058 | Yes |
| 20 | IGF2R |  |  | 470 | 0.444 | 0.4124 | Yes |
| 21 | TIMP3 |  |  | 516 | 0.428 | 0.4080 | Yes |
| 22 | PEA15 |  |  | 521 | 0.427 | 0.4221 | Yes |
| 23 | BNIP3L |  |  | 565 | 0.409 | 0.4179 | Yes |
| 24 | CFLAR |  |  | 569 | 0.408 | 0.4317 | Yes |
| 25 | DNAJC3 |  |  | 689 | 0.374 | 0.3918 | No |
| 26 | SC5D |  |  | 737 | 0.362 | 0.3841 | No |
| 27 | FAS |  |  | 740 | 0.361 | 0.3966 | No |
| 28 | SPTAN1 |  |  | 815 | 0.344 | 0.3760 | No |
| 29 | NEDD9 |  |  | 817 | 0.344 | 0.3883 | No |
| 30 | BCL2L1 |  |  | 949 | 0.317 | 0.3410 | No |
| 31 | GADD45A |  |  | 1028 | 0.305 | 0.3171 | No |
| 32 | CYLD |  |  | 1085 | 0.295 | 0.3027 | No |
| 33 | CTNNB1 |  |  | 1190 | 0.280 | 0.2662 | No |
| 34 | IFNGR1 |  |  | 1289 | 0.269 | 0.2319 | No |
| 35 | TAP1 |  |  | 1317 | 0.266 | 0.2296 | No |
| 36 | EMP1 |  |  | 1598 | -0.285 | 0.1137 | No |
| 37 | VDAC2 |  |  | 1779 | -0.353 | 0.0455 | No |
| 38 | TNFRSF12A |  |  | 2116 | -0.694 | -0.0805 | No |
| 39 | BRCA1 |  |  | 2138 | -0.758 | -0.0617 | No |
| 40 | HMGB2 |  |  | 2241 | -1.469 | -0.0532 | No |
| 41 | TOP2A |  |  | 2246 | -1.562 | 0.0032 | No |
Table: GSEA details [plain text format]

  

Fig 2: HALLMARK\_APOPTOSIS: Random ES distribution      
 Gene set null distribution of ES for **HALLMARK\_APOPTOSIS**
